# A Multiple Definitions Model of Classification Into Fuzzy Categories

**DOI:** 10.3389/fpsyg.2019.00944

**Published:** 2019-04-26

**Authors:** Thomas M. Gruenenfelder

**Affiliations:** Department of Psychological and Brain Sciences, Indiana University, Bloomington, IN, United States

**Keywords:** category representation, conceptual representation, human reasoning, fuzzy reasoning, fuzzy concepts, classification

## Abstract

This paper describes a new hypothesis, referred to as the multiple definitions model, concerning the mental representation of fuzzy concepts. The basic claim of the model is that such concepts are represented as a set of multiple definitions, where each definition is exact. Fuzziness results from the fact that using such concepts requires sampling multiple such exact definitions of the concept. The model was applied to concepts that can be defined as a range of values over a single dimension (such as middle-age), and tested using conjunctions and disjunctions of middle-age (e.g., “A person is middle-aged at both 50 and 63.”). The model predicts that, controlling for the truths of individual ages, the truths of conjunctions involving ages that are close together will be judged higher than the truths of conjunctions involving ages farther apart, and that the opposite effect will occur for disjunctions (the distance effect). The results of two experiments confirmed this prediction. However, both experiments also found that conjunctions were judged truer than the less true of their component ages, and that disjunctions were judged less true than the truer of their component ages. The model does not predict this “minimax” effect. One possible explanation of the minimax effect was tested; another modeled. The overall conclusion is that the multiple definitions model is a viable contender to explain the distance effect. The minimax effect, however, is still in need of a satisfactory explanation.

## Introduction

This paper is concerned with the mental representation of genuinely vague concepts, and particularly with such concepts that can be defined as a range of values in a one-dimensional or at least low dimensional space. Examples of such concepts are middle-age (the concept is defined as a range of ages), a fair wage for a plumber (the concept is defined as a range of hourly wages), and a Midwestern city in the United States (the concept is defined along an east–west and north–south extent). Multiple, mutually exclusive concepts can be defined in such spaces (young, middle-age, old), but without clear boundaries between the different concepts. For instance, both middle-aged and old can be defined as a range of ages, but there is no precise age that is the boundary between those two concepts.

The current paper describes a *multiple definitions model* of how such concepts are mentally represented. According to this model, a person has many definitions of each such concept, where each definition is a range of values on the dimension on which the concept is defined. For example, one definition of middle-age is from 42 years old to 54; another is from 44 to 62. Similarly, one definition of a fair wage for a plumber might be from $27.00 to $41.00 per hour; another might be from $19.00 to $23.00 per hour. Each definition is unambiguous, with clear, sharp boundaries. Given just one definition, a person of a given age is either middle-aged or not. Ambiguity, perhaps better stated as graded category membership (Rosch, 1975; Rosch and Mervis, 1975; McCloskey and Glucksberg, 1978; Hampton, 1979), results from the fact that a given person has multiple definitions of such concepts, each definition with different boundaries. When using, thinking about or making decisions regarding membership in such concepts, people do not rely on a single definition, but sample multiple definitions from their population of definitions and use some aggregate measure across that sample to determine their usage, thought, or decision. For instance, when determining whether a 45-year-old person is middle-aged, multiple definitions of middle-age are sampled and the truth of the proposition that a person is middle-aged at 45 is proportional to the number of definitions in the sample that contain 45.

It is not an accident that all the above examples are concepts that span a middle range of values along the dimension on which they are defined. Under minimal assumptions, such concepts provide a straightforward test of the multiple definitions model. That test in turn makes the model not one of just conceptual representation but also of how people reason with fuzzy, ill-defined concepts. Those minimal assumptions are that (1) each definition is a continuous range of values with no interruptions (“45–64” is a legitimate definition of middle-age; “45–47 and 50–64” is not), and 2) not all the definitions are identical.^1^ Under these assumptions, when reasoning about conjunctions or disjunctions of potential category exemplars, the model predicts what is referred to here as the *distance effect*.

Before explaining the distance effect, some notation is introduced. t(X) represents the judged truth of a simple proposition such as “A person is middle-aged at age X.” t(X ∧ Y) represents the truth of a conjunction involving two ages: “A person is middle-aged at both X and Y.” t(X ∨ Y) is the truth of a disjunction involving two ages: “A person is middle-aged at either X or Y or both.” Finally, P is used to indicate the peak middle-age, that age where t(P) is highest: t(P) ≥ t(X) for all ages X.

The distance effect is a consequence of straightforward processing assumptions made within the model concerning how people judge the truth of simple statements, conjunctions, and disjunctions. To determine the truth of a simple statement, a number of definitions are sampled and the truth is proportional to the proportion of definitions that include that age. To determine the truth of a conjunction, a number of definitions are also sampled, and the truth is proportional to the proportion of definitions that include both ages. Finally, to determine the truth of a disjunction, a number of definitions are again sampled, and the truth is proportional to the proportion of definitions that include one or the other or both ages.

Intuitively, all else being equal, because definitions are assumed to be continuous, two ages that are near to one another are more likely to be included in a given definition than two ages far apart. Hence, conjunctions involving two ages near one another are likely to be judged truer than conjunctions involving two ages far apart from one another. Conversely, for disjunctions, for ages that are near one another, most definitions that include one age also include the other—there are few that include only one or the other age. For ages farther apart, however, a definition that fails to include one age still has a relatively decent probability of including the other and hence contributing to the truth of the disjunction. Consequently, disjunctions are likely to be judged truer when they include two ages far apart from one another than when they include two ages near one another, all else being equal. That interaction is the distance effect: conjunctions involving near pairs of ages are likely to be judged truer than conjunctions involving far pairs of ages, whereas disjunctions involving far pairs of ages are likely to be judged truer than disjunctions involving near pairs of ages.

Somewhat more formally, consider three ages, A, X, and Y, t(X) = t(Y), X and Y are on opposites side of the peak middle age (P), A is on the same side of the peak as X, and A < X if X < P or A > X if X > P. As a concrete example, assume *P* = 50, A is 35, X is 40, and Y is 60, with t(X = 40) = t(Y = 60). Such a set of ages is referred to here as an *outside set*, since the age with the unique truth value (A) is outside the range of the two ages with the same truth value (40–60). X and Y are referred to as an *equal pair* of ages, since t(X) = t(Y).

Under the conditions just described, the two ages on the same side of the peak middle age (35 and 40 in the example) are referred to here as a *near* pair of ages; the two ages on opposite sides of the peak (35 and 60 in the example) are referred to as a *far* pair of ages. The distance effect is the prediction that a conjunction involving a near pair of ages will be judged to be truer than a *corresponding conjunction* involving a far pair of ages. A corresponding conjunction is defined to be one in which the truths of the two simple statements comprising the conjunction (“A person is middle-aged at X.” “A person is middle-aged at Y.”) are the same for the near and far pairs. In contrast, a disjunction involving a far pair of ages will be judged truer than a corresponding disjunction involving a near pair of ages. In the example, t(35 ∧ 40) will be higher than t(35 ∧ 60), but t(35 ∨ 60) will be higher than t(35 ∨ 40). Note that by choosing two ages such that t(X) = t(Y) (i.e., equal pairs of ages), and then pairing a third age with each of those two ages, the truths of the individual statements comprising near conjunctions (disjunctions) are the same as the truths comprising far conjunctions (disjunctions). That is, near and far compounds do not differ on the truth values of the individual ages comprising them.

To see the prediction of the distance effect for an outside set of ages, note that any definition that includes the far pair (35 and 60) must also include the near pair (35 and 40). Hence, t(A ^∧^ X) ≥ t(A ^∧^ Y). In our example, t(35 ^∧^ 40) ≥ t(35 ^∧^ 60). In addition, especially if A is sufficiently close to X, there are likely to be definitions that include A and X but not Y. In that case, the inequality becomes strict: t(A ^∧^ X) > t(A ^∧^ Y). For disjunctions, by definition, since t(X) = t(Y) the same number of definitions include X as include Y, and all those definitions contribute equally to any disjunction involving X or Y. t(A ∨ X) is further increased only by additional definitions that include A but not X. However, since definitions are assumed to involve a continuous range of ages, any such definition would also not include Y and hence also increase t(A ∨ Y) by the same amount. Hence, t(A ∨ X) ≤ t(A ∨ Y). Using our example ages, since t(40) = t(60) the same number of definitions include 40 as include 60, and all those definitions contribute equally to any disjunction involving 40 or 60. t(35 ∨ 40) is further increased only by additional definitions that include 35 but not 40. However, since definitions are assumed to involve a continuous range of ages, any such definition would also not include 60 and hence also increase t(35 ∨ 60) by the same amount. Hence, t(35 ∨ 40) ≤ t(35 ∨ 60). In addition, again especially if A is sufficiently close to X, there are likely to be definitions that include both A and X (and do not add to the truth of their disjunction above and beyond the definitions that include X) but do not include Y (and hence do add to the truth of the disjunction involving A and Y). In that case, the inequality again becomes strict: t(A ∨ X) < t(A ∨ Y).

A distance effect is also likely, though not necessarily guaranteed, to occur for *inside sets* of ages. An inside set is defined here to be the case where the age with the unique truth value (A) is inside the range of the two ages with the same truth value. Continuing with our example ages from above, changing A from 35 to 45 would make the set an inside set. Under most reasonable assumptions regarding how definitions are distributed, more definitions will include 40 and 45 than will include 45 and 60. However, it is technically possible that the truth of a far conjunction (45 and 60) could be higher than the truth of a near conjunction (40 and 45). Such would be the case if many of the definitions involving 60 were wide and included 45 (e.g., 42–62; 44–65; 43–61) but those involving 40 were narrow and failed to include 45 (e.g., 38–41; 39–44; 40–43). However, given the way near and far pairs were constructed in the present study (see the “Data Analysis” section of Experiment 1), corresponding conjunctions and disjunctions come in pairs. Given the age A on one side of the peak, there would be a second age, A′, on the opposite side of the peak, that could also be paired with X and Y to form near and far pairs. Now, however, A′ and Y would be the near compound and A′ and X the far compound. The age 55 would work in our example. That age A′ would be included in those wide definitions that include Y (the age 60 in our example), thereby increasing the truth of the near conjunction A′ ^∧^ Y (50 ^∧^ 60). However, A′ would not be included in the narrow definitions that include X (the age 40 in the example), hence decreasing the truth of the far conjunction A′ ^∧^ X (40 ^∧^ 55). Hence, even for inside ages, near conjunctions are likely to be rated as truer than far conjunctions. A converse argument can be made that far disjunctions, even for inside sets of ages, will tend to be rated as truer than near disjunctions. Consider the same (seemingly unlikely) scenario as described above for conjunctions—definitions that include Y (60) are unusually wide and hence also include A (45) while those that include X (40) are unusually narrow and hence exclude A (45). In such a case, the near disjunction (X ∨ A) (40 ∨ 45) could be rated as true or truer than the far conjunction (Y ∨ A) (60 ∨ 45), an outcome opposite to the predicted distance effect. However, when A′ (55) is also considered, the predicted distance effect would emerge. Definitions that include Y (60) are wide and hence would also tend to include A′ (55). Since they already include Y, however, the inclusion of A′ in the definition would do nothing to raise the truth value of the near disjunction A′ ∨ Y (55 ∨ 60). In contrast, those same wide definitions that include A′ (55) would tend to exclude X (40), while the narrow definitions that include X would tend not to include A′. Consequently, both those subsets of definitions raise the truth value of the far disjunction X ∨ A′ (40 ∨ 55), resulting in the far disjunction (X ∨ A′) (40 ∨ 55) being rated as truer than the near disjunction (A′ ∨ Y) (55 ∨ 60).

In brief, the distance effect is all but guaranteed to occur for outside sets of ages and is very likely to occur for inside sets of ages.

The predictions of the multiple definitions model can be contrasted with those of three other rules for determining the truths of compound statements with graded truth values. According to the product rule, based on the work of Goguen (1969), the truth of a conjunction is simply the product of the truth of its constituents: t(X ^∧^ Y) = t(X)t(Y). The truth of a disjunction is the sum of the truths of the constituents minus the product of their truths: t(X ∨ Y) = t(X) + t(Y) – t(X)t(Y). The minimum/maximum rule is based on the work of Zadeh (1965). Here, the truth of the conjunction is equal to the minimum truth of the constituents, t(X ^∧^ Y) = min[t(X),t(Y)], and the truth of a disjunction is equal to the maximum truth of the constituents, t(X ∨ Y) = max[t(X),t(Y)]. Finally, an averaging rule was considered, in which the truth of both conjunctions and disjunctions is simply the average of the truths of the constituents, t(X ^∧^ Y) = t(X ∨ Y) = mean[t(X),t(Y)].

Why did the present experiments use equal pairs of ages, as defined above? Oden (1977) compared the ability of the product and minimum/maximum rules to predict peoples’ ratings of the truth of various conjunctions and disjunctions using the concepts of *birds* and *furniture*. In general, he found better fits to the product rule than to the minimum/maximum rule. As he noted, however, there are situations where intuitively the minimum/maximum rule makes more sense than the product rule. Hence, an experimental design that can distinguish both these rules from the multiple definitions model is desired. In the case of all three of the above rules (minimum/maximum, product, and averaging), the truth of a compound can be determined entirely by examining the truths of its constituents. That is, they conform to the Simple Functional Hypothesis (SFH) (Osherson and Smith, 1982), in which t(X ^∧^ Y) = f[t(X), t(Y)], where f is some function, and similarly for disjunctions. Unlike the case for the multiple definitions model, once the truth of the simple statements is determined, there is no further need to consider the actual ages involved in the compound. Only the truth values of the simple statements comprising the compound matter when evaluating the truth of the compound. Consequently, *when the compounds are based on equal pairs of ages*, none of these models predicts a distance effect, since the truth values of the simple statements comprising the compounds are the same for the near and far ages. More generally, *provided that equal pairs of ages are used*, then finding a distance effect is inconsistent with not only the product, minimum/maximum, and averaging rules. It is inconsistent with any rule that conforms to the SCH. Finally, by using equal pairs of ages, any potential confound between truth value and pair distance is avoided.

The multiple definitions model is quite similar to Kamp and Partee’s (1995) approach to explaining graded category membership using completions of partial models. Briefly, as applied to middle-age, that approach begins with a range of ages that are all definitely to be considered middle-age. For the sake of an example, suppose that range of ages is 45–55. A second set of ages includes ages that are considered to be definitely not middle-age. For the sake of the example, suppose that those are all ages under 35 and all ages over 65. A third set of ages (36–44 and 56–64) has an indeterminate status in the category middle-age. The partial model is then completed by defining additional sets of ages that are to be considered middle-age by extending the original definition of middle-age downward 1 year at a time (44–55, 43–55, and so on), upward 1 year at a time (45–56, 45–57, and so on), 1 year downward and 1 year upward at a time (44–56, 43–57, and so on), etc.—all additional possible combinations of the indeterminate ages are considered that preserve the linear ordering of ages (i.e., just as in the multiple definitions model, split ranges of ages are not permitted—45–55 and 62–64 is not a valid completion of the model, though 45–64 is). The degree of membership of a particular age in the category middle-age is then simply the proportion of completions that contain that age. Similarly, the degree that two ages are both to be considered middle-age would be the proportion of completions that include both ages. The degree that one or the other or both of two ages are to be considered middle-age would be the proportion of completions that include one or the other or both. The completions play a similar role in the model as do the definitions in the multiple definitions model.

Kamp and Partee’s (1995) model in turn has been extended by Douven (2016) and Douven et al. (2017), using Gärdenfors’ (2000) concept of convex regions in conceptual spaces, to apply to concepts defined in a space with an arbitrarily high number of dimensions. Likewise, the multiple definitions approach can be extended to concepts defined in higher dimensional spaces by using definitions that are *n*-dimensional volumes rather than simple one-dimensional ranges. Although there are some differences, mentioned in the “General Discussion” section, between the approaches of Kamp and Partee (1995), Douven (2016), and Douven et al. (2017), on the one hand, and the multiple definitions approach on the other hand, the two approaches make the same qualitative predictions concerning the present experiments. Hence, to the extent that the results of the present experiments support the multiple definitions model, they also support the models of Kamp and Partee (1995), Douven (2016), and Douven et al. (2017).

Experiment 1 tests whether, under the appropriate experimental conditions, the distance effect predicted by the multiple definitions model does in fact occur. That is, are near conjunctions, as defined above, rated as truer than far conjunctions, and near disjunctions rated as less true than far disjunctions. Experiment 2 is an opportunity to replicate the results of Experiment 1 with respect to the distance effect. In addition, it explores an unexpected finding from Experiment 1 that is not consistent with the multiple definitions model. Finally, the results of a simulation of an expanded multiple definitions model that is intended as another possible explanation of that unexpected finding are reported.

## Experiment 1

### Methods

#### Participants

Thirty-one Introductory Psychology students from Indiana University participated in this experiment in partial fulfillment of a course requirement. The Indiana University Institutional Review Board approved the study. All participants provided written informed consent in accordance with the Declaration of Helsinki.

#### Stimuli

Stimuli consisted of simple sentences, (“A person is middle-aged at X.”), conjunctions (“A person is middle-aged at both X and Y.”), and disjunctions (“A person is middle-aged at either X or Y or both.”). Ages used in the simple sentences were 15, 25, 30, 33, 37, 40, 45, 50, 53, 57, 60, 65, and 75. This range of ages was chosen as pilot work indicated that it was more than sufficient to encourage participants to use the entire rating scale (Parducci, 1965). Ages used in compound sentences (conjunctions and disjunctions) were all pair-wise combinations of those same ages from 25 through 65 inclusive. In the compound sentences, the younger age was always presented first (“A person is middle-aged at both 45 and 53,” but not “A person is middle-aged at both 53 and 45.”). Pilot testing using sentences with a similar grammatical structure as the compounds but involving non-fuzzy concepts (e.g., “Cities in Indiana include both Bloomington and Indianapolis,” “Cities in Indiana include either Indianapolis or Chicago or both.”) indicated that participants did indeed interpret the conjunctive sentences as logical conjunctions and the disjunctive sentences as inclusive logical disjunctions.

#### Design and Procedure

All experimental factors were varied within-subjects. That is, all participants rated all sentences, both the simple and compound. Further, all the sentences, both simple and compound, were intermixed with one another and presented in a random order (with the restriction that a sentence could not be followed by an identical sentence). Such a within-subject design avoids problems of interpretation that can occur when different participants rate different sentences, as those different groups may use the rating scale differently in order to spread their responses across the entire scale. Similarly, intermixing all sentences in a single experimental session, as opposed to blocking the presentations by sentence type, avoids problems of the same participant potentially using the rating scale differently on different occasions. Sentences were presented one-at-a-time, centered horizontally and vertically on a computer monitor, black print on a white background. Participants were instructed to rate the truth of each sentence on a scale from 1 (very false) to 6 (very true) by pressing a button on a 6-button response box. The response buttons were arranged horizontally, with the left-most button labeled “Very False” and the right-most button labeled “Very True.” Participants were told that although there was no time pressure for making their judgments, their first impression was often best. Each of the simple sentences was presented three times for rating. Each compound was presented once. The sentence remained on the screen until the participant made a response. It was then erased and the next sentence followed a 0.5 s inter-stimulus interval. The order of sentences was randomized separately for each participant.

#### Data Analysis

The test of the prediction of the distance effect requires finding, for each individual participant, two ages, X and Y, such that X < P < Y (recalling that P is the peak middle-age) and t(X) = t(Y). Accordingly, the truth rating assigned to each simple statement by each participant was first determined by averaging the ratings of that participant across the three presentations of each simple statement. P was then simply the age that received the maximum such rating. For participants where more than one age received that maximum, the peak was defined as extending from the lowest such age through the highest such age, inclusive. For each participant, I then searched for two ages, X and Y, such that t(X) = t(Y), where t(X) is the mean truth rating assigned by that participant across the three simple statements involving X, and X < P < Y. For 8 of the 31 participants, no such equal pairs occurred and these participants were hence dropped from the analysis, leaving data from 23 participants.

Near pairs were defined to be those pairing X with the age A that was immediately above or below X in the sequence of ages used, subject to the constraint that A actually be used in compound sentences (If X were 25, then one value of A would be 15, but no compounds involved the age 15.) and to the constraint that A not be the peak middle age. Corresponding far pairs were then defined as those involving A and Y. Similarly, near pairs involving Y were defined as those pairing Y with the age B immediately above or below it in the sequence of ages used, subject to the same constraints involving A and X. The corresponding far pairs were defined as those involving X and B.

For example, suppose that for a particular participant, *P* = 50, and t(37) = t(60) < t(*P*). Then the near pairs of ages were (33, 37), (37, 40), (57, 60), and (60, 65). The corresponding far pairs of ages were (33, 60), (40, 60), (37, 57), and (37, 65). Note that the set of ages 33, 37, and 60 and the set of ages 37, 60, and 65 form outside sets, and the set 37, 40, and 60 and the set 37, 57, and 60 form inside sets.

### Results

The results shown and reported here are for a combined analysis that included both inside and outside sets of ages. An analysis was also conducted that included only outside sets. The results of the two analyses were very similar, the one exception being that the distance effect for disjunctions was somewhat larger in the analysis that included only outside sets of ages.

**FIGURE 1:**
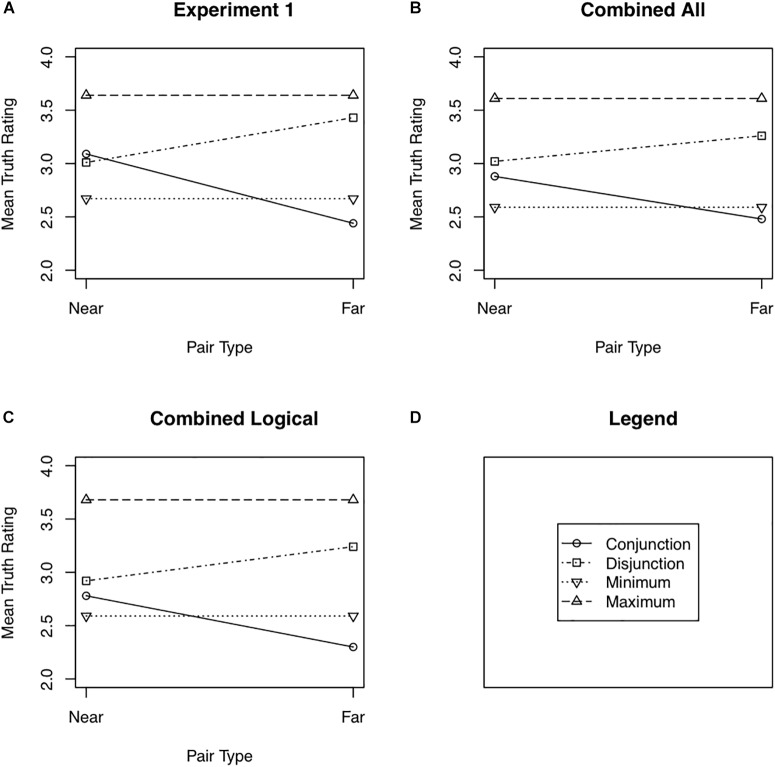
Mean truth ratings as a function of pair type (near or far) for Experiment 1 **(A)**, the combined analysis of Experiment 1 and all participants from Experiment 2 **(B)**, and the combined analysis of Experiment 1 and the Logical participants from Experiment 2 **(C)**. Panel **(D)** shows the legend used in Panels **(A)** through **(C)**.

Figure 1A shows the mean truth rating to near and far conjunctions and disjunctions. The minimum and maximum of the truths of the two simple statements comprising each conjunction and disjunction are also shown in Figure 1A. These means along with their 95% confidence intervals are also shown in Table 1. Note that the way near and far pairs were defined (see the “Data Analysis” section of Experiment 1) results in the minimum necessarily being the same for near and far pairs, and likewise for the maximum.

**Table 1:** Mean truth ratings from 1 (very false) to 6 (very true) for near and far conjunctions and disjunctions and to the less true (minimum) and more true (maximum) constituent.

Conjunctions		Disjunctions		Minimum	Maximum
Near	Far	Near	Far		
**Experiment 1**					
3.09	2.44	3.01	3.43	2.67	3.64
(2.49–3.69)	(1.92–2.95)	(2.49–3.54)	(2.81–4.05)	(2.13–3.20)	(3.14–4.14)
**Experiment 2: All**					
2.77	2.50	3.01	3.17	2.40	3.59
(2.47–3.07)	(2.18–2.82)	(2.72–3.31)	(2.82–3.52)	(2.15–2.64)	(3.33–3.85)
**Experiment 2: Averaging**					
3.18	3.00	3.28	3.34	2.39	3.77
(2.68–3.68)	(2.39–3.61)	(2.71–3.85)	(2.78–3.90)	(1.93–2.85)	(3.26–4.28)
**Experiment 2: Logical**					
2.51	2.19	2.85	3.07	2.40	3.48
(2.15–2.87)	(1.86–2.51)	(2.52–3.18)	(2.60–3.54)	(2.09–2.17)	(3.17–3.78)
**Combined: All**					
2.88	2.48	3.01	3.26	2.59	3.61
(2.60–3.16)	(2.21–2.74)	(2.76–3.27)	(2.96–3.57)	(2.35–2.83)	(3.37–3.84)
**Combined: Logical**					
2.78	2.30	2.92	3.24	2.59	3.68
(2.44–3.11)	(2.02–2.58)	(2.63–3.22)	(2.86–3.61)	(2.31–2.88)	(3.39–3.97)

*Numbers in parentheses are 95% confidence intervals.*

The predicted distance effect is an interaction of Question Type (conjunction vs. disjunction) with Pair Distance (near pairs vs. far pairs). A 2 (Question Type) × 2 (Pair Distance) repeated measures analysis of variance found strong support for this interaction, *F*(1,22) = 13.66, Cohen’s *d* = 0.74, unit information Bayes factor (Rouder et al., 2009), the likelihood ratio of the alternative to the null, = 41.82.^2^ Near conjunctions were rated as truer than far conjunctions, *t*(22) = 3.63, Cohen’s *d* = 0.76, Bayes factor favoring the alternative over the null = 36.43. Although there was a tendency for far disjunctions to be rated as truer than near disjunctions, the data were more variable and the effect was much weaker than for conjunctions, *t*(22) = −1.76, Cohen’s *d* = 0.37, Bayes factor favoring the alternative over the null = 1.14. Fifteen of the 23 participants rated near conjunctions as truer than far conjunctions, five rated near and far conjunctions as equally true, and three rated far conjunctions as truer than near conjunctions. Thirteen participants rated far disjunctions as truer than near disjunctions, five rated far and near disjunctions as equally true, and five rated near disjunctions as truer than far disjunctions.

Although the finding of a distance effect for near and far pairs for conjunctions and disjunctions is predicted by the multiple definitions model, other aspects of the data are at odds with that model. According to that model, the truth of a conjunction must be no greater than the minimum truth of the two simple statements comprising that conjunction: t(X ^∧^ Y) ≤ min[t(X),t(Y)]. Only definitions that include both ages contribute to the truth of the conjunction. These definitions also contribute to the truth of each component statement. In addition, there may be other definitions, that include only one of the two ages, that contribute to the truth of the simple statement but not to the truth of the conjunction. Similarly, the truth of a disjunction must be greater than or equal to the maximum truth of the two simple statements comprising that disjunction, t(X ∨ Y) ≥ max[t(X),t(Y)]. All definitions that include X contribute to the truth of the disjunction, but so do those definitions that include Y but not X. The data violate these constraints. There is a tendency, albeit a weak one, for near conjunctions to be rated as truer than the minimum, as can be seen in Figure 1A, *t*(22) = 1.98, Cohen’s *d* = 0.35, Bayes factor favoring the alternative over the null = 1.58. There is a strong tendency for near disjunctions to be rated as less true than the maximum, *t*(22) = −4.61, Cohen’s *d* = 0.93, Bayes factor favoring the alternative over the null = 290.83. I refer to the tendency of near conjunctions to be rated as truer than the minimum as the *minimum effect*,^3^ the tendency of near disjunctions to be rated as less true than the maximum as the *maximum effect*, and the two effects taken together as the *minimax effect*. For near conjunctions, 14 participants showed the minimum effect, 3 assigned the same mean truth rating to near conjunctions and the minimum constituent, and 6 rated the minimum constituent as truer than the near conjunction. For near disjunctions, 19 participants showed the maximum effect, and 4 rated near disjunctions as truer than the maximum constituent.

The use of analyses of variance on rating scale data is not without controversy, since it is not necessarily the case that participants use the rating scale in such a way that the data reflect an interval, as opposed to merely ordinal, scale. (An advantage of being able to use analyses of variance is that higher order interactions can be tested, as was done in Experiment 2.) The methods used in the present experiment do meet minimal criteria that have been delineated for treating rating scale data as an interval measurement (Harpe, 2015). Nevertheless, the data were also analyzed using non-parametric methods. The proportion of participants rating near conjunctions as truer than far conjunctions (0.652) was greater than the proportion rating near disjunctions as truer than far disjunctions (0.217), *z* = 2.97, *p* < 0.005, indicating an interaction of Question Type with Pair Distance. Friedman Tests for Repeated Measures found reliably higher truth ratings to near conjunctions than to far conjunctions, χ^2^_r_ (1, *N* = 23) = 6.260, *p* < 0.025, a trend toward higher ratings to far disjunctions than near disjunctions, χ^2^_r_ (1, *N* = 23) = 2.782, *p* = 0.095, a trend toward near conjunctions being rated as truer than the minimum constituent, χ^2^_r_ (1, *N* = 23) = 2.782, *p* = 0.095, and reliably higher truth ratings to the maximum constituent than to near disjunctions, χ^2^_r_ (1, *N* = 23) = 9.782, *p* < 0.025. This general pattern of the non-parametric tests paralleling the analyses of variance also held for Experiment 2 and for the combined analysis discussed in connection with Experiment 2.

### Discussion

Experiment 1 found good evidence for the distance effect. Near conjunctions were rated as truer than far conjunctions, whereas far disjunctions tended to be rated as truer than near disjunctions. This effect is predicted by the multiple definitions model, but not by the product, minimum/maximum, or averaging rules, or any other rule that conforms to the SFH. The experiment also found good evidence of a minimax effect—near disjunctions were rated as less true than the maximum truth of the simple component statements, and near conjunctions tended to be rated as more true than the minimum component. Though the distance effect is predicted by the multiple definitions model, the minimax effect clearly is not. The minimax effect is also inconsistent with the product and the minimum/maximum rules.

The minimax effect is consistent with a simple averaging model, where participants determine the truth of a compound statement by averaging the truths of the two simple statements comprising that compound. In its simplest form, t(X ^∧^ Y) = t(X ∨ Y) = mean[t(X),t(Y)] (where the truths of the simple statements might still be determined by a process akin to sampling multiple definitions). Note, however, that such an averaging model does not predict the distance effect, and hence does not explain the entire pattern of results of Experiment 1. One possibility is that different participants use different strategies when determining the truth of compound statements. Some may simply average the truths of the component statements. Those participants would show a minimax effect but not a distance effect. Indeed, Oden (1977) found that at least for conjunctions, the data from a minority of his participants were better fit by an averaging rule than either the product or minimum/maximum rules (see also Smith and Osherson, 1984). Other participants may determine the truth of compounds following the sampling procedure proposed in the multiple definitions model. Those participants would show a distance effect but not a minimax effect. Experiment 2 tested this hypothesis by adding a third type of compound that was intended to identify those participants using an averaging strategy and those perhaps following the multiple definitions sampling procedure.

## Experiment 2

Experiment 2 served two purposes. The first was simply to replicate the primary effects of Experiment 1. The second was to test the hypothesis described above, that the minimax effect was due to some proportion of participants using an averaging strategy.

Experiment 2 used the same simple and compound sentences used in Experiment 1, but added conjunctions involving three ages, referred to here as triple conjunctions. The triple conjunctions were added in an effort to distinguish participants who determined the truth of compound sentences using an approach akin to the multiple definitions model from participants who determined the truth of compounds by averaging together the truths of the simple sentences comprising the compound. Consider three ages, X < Y < Z where X < P (the peak middle-age) < Z, t(Y) > t(X), and t(Y) > t(Z). Now consider the relative magnitudes of the truths of t(X ^∧^ Z) and t(X ^∧^ Y ^∧^ Z), as predicted by the multiple definitions model and by an averaging model. According to the multiple definitions model, t(X ^∧^ Z) = t(X ^∧^ Y ^∧^ Z) since the set of definitions that include X and Z must be precisely the set that includes X, Y, and Z. An averaging model, on the other hand, predicts that t(X ^∧^ Y ^∧^ Z) > t(X ^∧^ Z), since mean[t(X), t(Y), t(Z)] > mean[(t(X), t(Z)], given that t(Y) > t(X) and t(Y) > t(Z). Hence, by comparing the performance of participants on these triple conjunctions, those participants following a multiple definitions strategy and those following an averaging strategy could potentially be identified. Those following the multiple definitions strategy are predicted to show a distance effect but no minimax effect. Those following an averaging strategy are predicted to show no distance effect but to show the minimax effect.

### Methods

#### Participants

Eighty-four Indiana University Introductory Psychology students participated in this experiment in partial fulfillment of a course requirement.^4^ The Indiana University Institutional Review Board approved the study. All participants provided written informed consent in accordance with the Declaration of Helsinki.

#### Stimuli

The same set of simple statements, conjunctions (re-worded as described below) and disjunctions used in Experiment 1 were used in Experiment 2. In addition, a triple conjunction, involving three ages, was formed from each of the original double conjunctions, involving two ages, by adding as the third age the age halfway between the two ages in the double conjunction, rounded to the nearest whole age. For example, from the double conjunction involving the ages 33 and 57, the triple conjunction involving ages 33, 45, and 57 was formed. In addition, in order to allow for a more natural wording of the triple conjunctions, the wording of double conjunctions was changed to, “A person is middle-aged at both of these ages: 33 and 57.” Triple conjunctions were worded as, “A person is middle-aged at all three of these ages: 33, 45, and 57.”

#### Design and Procedure

The design and procedure followed that of Experiment 1. All sentences were rated by all participants and all the sentences were intermixed with one another. Sentences were presented one-at-a-time, centered horizontally and vertically on a computer monitor. Participants rated the truth of each sentence on a scale from 1 (very false) to 6 (very true) by pressing a button on a six-button response box. Each of the simple sentences was presented three times for rating. Each compound was presented once. The sentence remained on the screen until the participant made a response. It was then erased and the next sentence followed a 0.5 s inter-stimulus interval. The order of sentences was randomized separately for each participant.

#### Data Analysis

As in Experiment 1, the data analysis required finding on an individual participant basis equal pairs of ages, i.e., two ages X and Y, with X being younger than the peak middle age, Y being older than the peak middle age, and t(X) = t(Y). One or more such equal pairs were found for 44 of the participants and only these participants were included in the subsequent analyses. These 44 participants were then divided into what are here referred to as Logical participants and Averaging participants based on their performance on the triple conjunctions relative to the corresponding double conjunctions. First, for each of those 44 participants, all triple conjunctions (A ^∧^ B ^∧^ C) were identified for which A < B < C, t(B) > t(A) and t(B) > t(C). That is, A and C formed the lower and upper bound of the range spanned by the three ages, and B had the highest truth rating in the simple statements involving the three ages. For each individual participant, across all such sets of three ages, the mean truth rating given to the triple conjunction, t(A ^∧^ B ^∧^ C), was compared to the mean truth rating given to the double conjunction containing the youngest and oldest age in that range, t(A ^∧^ C). Note that according to the multiple definitions model, t(A ^∧^ B ^∧^ C) = t(A ^∧^ C) since all definitions that include both A and C also include B. According to the averaging rule, in contrast, t(A ^∧^ B ^∧^ C) > t(A ^∧^ C) since t(B) > t(A) and t(B) > t(C). Hence, if a participant’s mean truth ratings to triple conjunctions was higher than the mean rating to corresponding double conjunctions, the participant was assigned to the Averaging group. Otherwise the participant was assigned to the Logical group. By this criterion, 17 participants were assigned to the Averaging group and 27 to the Logical group.

### Results

**FIGURE 2:**
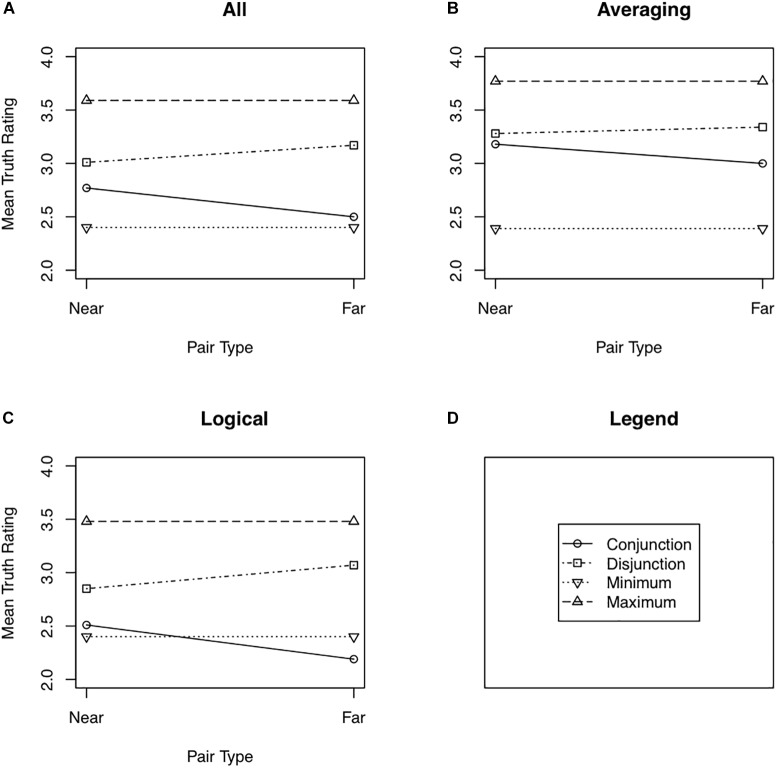
Mean truth ratings as a function of pair type (near or far) for all participants from Experiment 2 **(A)**, the Averaging participants from Experiment 2 **(B)**, and the Logical participants from Experiment 2 **(C)**. Panel **(D)** shows the legend used in Panels **(A)** through **(C)**.

Figure 2 shows the mean truth ratings assigned to near and far pairs for (double) conjunctions and disjunctions as well as the maximum and minimum truth rating assigned to the two simple statements comprising each conjunction and disjunction. These means along with their 95% confidence intervals are also shown in Table 1. The separate panels show the data combined across all 44 participants for whom equal pairs were found (Figure 2A), for just the Averaging group (Figure 2B), and for just the Logical group (Figure 2C). Recall that the multiple definitions model predicts a distance effect but no minimax effect, whereas the averaging model predicts a minimax effect but no distance effect.

The Logical group showed a moderate distance effect, *F*(1,26) = 6.88, for the Question Type × Pair Distance interaction, Cohen’s *d* = 0.50, Bayes factor = 4.89 favoring the alternative hypothesis. Near conjunctions tended to be rated as truer than far conjunctions, *t*(26) = 2.06, Cohen’s *d* = 0.40, Bayes factor = 1.74 favoring the alternative hypothesis. The converse effect for disjunctions was weaker, *t*(26) = 1.56, Cohen’s *d* = 0.30, with the Bayes factor (1.24) actually slightly favoring the null hypothesis. Fifteen of the 27 Logical participants rated near conjunctions as truer than far conjunctions, five rated them as equally true, and seven rated far conjunctions as truer than near conjunctions. For disjunctions, 6 participants rated near disjunctions as truer than far disjunctions, 6 rated the two types of pairs as equally true, and 15 rated far disjunctions as truer than near disjunctions. There was little evidence of a minimum effect in the Logical group, with near conjunctions being rated as only slightly truer than the minimum of the constituent sentences, *t*(26) = 0.81, Cohen’s *d* = 0.16, Bayes factor = 2.77 favoring the null hypothesis. Twelve participants rated near conjunctions as truer than the minimum constituent, 2 assigned the same truth ratings to the near conjunctions and the minimum constituent, and 13 rated the minimum constituent as truer than the near conjunction. In contrast, there was stronger evidence of a maximum effect, with far conjunctions being rated as less true than the maximum of the constituent sentences, *t*(26) = 2.76, Cohen’s *d* = 0.53, Bayes factor = 6.50 favoring the alternative hypothesis. Nineteen of the Logical participants rated far disjunctions as less true than the maximum constituent, one assigned the same truth ratings to far disjunctions and the maximum constituent, and seven assigned a higher truth rating to the maximum constituent than to the far disjunction. The effect was stronger for near disjunctions.

The Averaging group showed a somewhat different pattern with respect to the distance effect and the minimum effect. The overall distance effect was quite weak, *F*(1,16) = 0.462, Cohen’s *d* = 0.16, Bayes factor = 2.48 favoring the null hypothesis. There was little evidence that near conjunctions were rated as truer than far conjunctions, *t*(16) = 0.63, Cohen’s *d* = 0.15, Bayes factor = 2.55 favoring the null, or that far disjunctions were rated as truer than near disjunctions, *t*(16) = 0.33, Cohen’s *d* = 0.08, Bayes factor = 2.92 favoring the null hypothesis. It is the case, however, that a relatively large proportion (12 of 17) of the Averaging participants did rate near conjunctions as truer than far conjunctions, with five rating far conjunctions as truer than near conjunctions. Eight participants rated far disjunctions as truer than near disjunctions, two rated the two types of disjunctions as equally true, and seven rated near disjunctions as truer than far disjunctions. In contrast to the Logical group, the Averaging group showed a robust minimum effect for both near and far conjunctions, *t*(16) = 3.18, Cohen’s *d* = 0.91, Bayes factor = 32.16 favoring the alternative hypothesis for near conjunctions; *t*(16) = 3.00, Cohen’s *d* = 0.72, Bayes factor = 8.84 favoring the alternative hypothesis for far conjunctions. For near conjunctions, 15 of the 17 participants showed a minimum effect and two showed the opposite. For far conjunctions, 12 participants showed the minimum effect, 4 showed the opposite effect, and 1 showed no difference between far conjunctions and the minimum constituent. The Averaging group also showed a maximum effect for both near and far disjunctions, *t*(16) = 2.28, Cohen’s *d* = 0.55, Bayes factor = 2.62 favoring the alternative hypothesis for near disjunctions; *t* = 2.78, Cohen’s *d* = 0.67, Bayes factor = 6.06 favoring the alternative hypothesis for far disjunctions. Four participants rated near disjunctions as truer than the maximum constituent and 13 rated the maximum constituent as truer. Three participants rated far disjunctions as truer than the maximum constituent and 14 rated the maximum as truer than the disjunction.

Although the above analyses suggest a larger distance effect in the Logical group than in the Averaging group, an overall analysis that included Group (Logical vs. Averaging) as a factor, found little evidence that the Question Type × Pair Distance interaction was modulated by Group, *F*(1,42) = 0.61, Cohen’s *d* = 0.24, Bayes factor = 1.96 favoring the null hypothesis. Similarly, Group did not modulate the maximum effect for disjunctions, a result not surprising given that both groups showed a large maximum effect. Group did modulate the minimum effect for near conjunctions, with the effect being larger in the Averaging group than in the Logical group, *F*(1,42) = 7.78, Cohen’s *d* = 0.86, Bayes factor = 8.25 favoring the alternative.

#### Combined Analyses of Experiments 1 and 2

Two analyses combining the data from Experiments 1 and 2 were conducted. The first included all participants from Experiment 1 (for whom equal pairs were found) and all participants from Experiment 2 (also for whom equal pairs were found). This analysis is referred to as the Combined All analysis. The second included the same participants from Experiment 1 but only the participants in the Logical group from Experiment 2. This analysis is referred to as the Combined Logical analysis. The mean truth ratings for both the Combined All (Figure 1B) and Combined Logical (Figure 1C) analyses to near and far disjunctions as well as the minimum and maximum truth ratings of the constituent simple sentences are shown in Figure 1. These means along with their 95% confidence intervals are also shown in Table 1.

The Combined All analysis showed a robust distance effect, for the Question Type × Pair Distance interaction, *F*(1,66) = 16.23, Cohen’s *d* = 0.49, Bayes factor = 206 favoring the alternative hypothesis. A strong distance effect occurred for conjunctions, *t*(66) = 3.49, Cohen’s *d* = 0.43, Bayes factor = 40.27 favoring the alternative hypothesis. The distance effect was weaker for disjunctions, *t*(66) = −2.26, Cohen’s *d* = 0.28, Bayes factor = 1.92 favoring the alternative hypothesis. The Combined All analysis also showed a minimum effect. Near conjunctions were rated as truer than the constituent with the minimum truth rating, *t*(66) = 2.66, Cohen’s *d* = 0.32, Bayes factor = 4.54 favoring the alternative hypothesis. Likewise, a maximum effect occurred. Near disjunctions were rated as less true than the constituent with the maximum truth rating, *t*(66) = −4.48, Cohen’s *d* = 0.55, Bayes factor = 925 favoring the alternative hypothesis.

The Combined Logical analysis showed a similar distance effect only stronger, *F*(1,49) = 19.90 for the Question Type × Pair Distance interaction, Cohen’s *d* = 0.63, Bayes factor = 666 favoring the null hypothesis. A strong distance effect occurred for conjunctions, *t*(49) = 3.96, Cohen’s *d* = 0.56, Bayes factor = 153 favoring the alternative hypothesis. The distance effect was again weaker for disjunctions, *t*(49) = −2.35, Cohen’s *d* = 0.33, Bayes factor = 2.54 favoring the alternative hypothesis. The data also showed little evidence of a minimum effect, with near conjunctions being rated only slightly truer than the constituent with the minimum truth rating, *t*(49) = 1.48, Cohen’s *d* = 0.21, Bayes factor = 1.81 favoring the null hypothesis. The disjunctions did show a maximum effect, with near disjunctions being rated as less true than the constituent with the maximum truth rating, *t*(49) = 5.40, Cohen’s *d* = 0.76, Bayes factor = 13077 favoring the alternative hypothesis.

### Discussion

The results of Experiment 2 provide some support for the hypothesis that different groups of people used different strategies to judge the truths of the conjunctions and disjunctions in that experiment. One group, identified as the Logical group, produced a robust distance effect and only, at best, a weak minimum effect, a pattern of results consistent with the multiple definitions model. The second group, identified as the Averaging group, produced at best a weak distance effect and a strong minimax effect, a pattern of results consistent with an averaging strategy. Two results, however, argue against the mixed-strategy hypothesis. First, in a combined analysis, group (Averaging versus Logical) did not strongly modulate the distance effect. Second, even in the Logical group, a strong maximum effect occurred for disjunctions, a result that should not occur if this group were strictly following the multiple definitions model.

The pattern, particularly given the maximum effect in the Logical group, does suggest a need for a model that can simultaneously produce a distance effect, a maximum effect, and possibly a minimum effect. One possible such model, and it is clearly and admittedly *post hoc*, retains the core assumption of the multiple definitions model, i.e., that the truths of statements like those used in the present study are evaluated by sampling multiple definitions that together represent the meaning of the concept. The two other important aspects were motivated by the following intuitions. First, the disjunctive “or” seems to have a connotative meaning of exclusion. “Either Charlie is a liar or Sam is a liar” seems to suggest that if Charlie is the liar than Sam is not. “A person is middle-aged at either 45 or at 60” similarly seems to suggest that at the other age they are not middle-aged. In terms of the multiple definitions model, the suggestion here is when evaluating a disjunction, the exclusionary connotative meaning of “or” causes people to contract the sampled definitions. If the definition sampled is, for example, “35 through 47,” it contracts to “39 through 43.” The overall effect is to lower the truth of disjunctions, relative to what would be expected based on the truths of the component statements, perhaps to the point where the truth of the disjunction is less than the maximum truth of its components.

In contrast, “and” seems to have a connotative implication of inclusion. “Both Wilbur Wood and Nolan Ryan were great pitchers (apologies to those not familiar with baseball),” suggests a definition of a good pitcher that includes both hard throwers like Ryan as well as slow, floating knuckleballers like Wood—a very expansive definition. A statement like, “A person is middle-aged at both 40 and at 70” suggests a very broad notion of middle-aged. This inclusive connotative meaning of “and” causes people to expand the sampled definitions of middle age when evaluating a conjunction. The definition “35 through 47” becomes “33 through 49.” The overall effect would be to increase the truth of the conjunction, perhaps to the point where it exceeds the minimum truth of its components.

I refer to these two ideas together as the contraction–expansion hypothesis. This hypothesis is at least somewhat similar to an *anchoring* hypothesis considered (and rejected) by Hampton (1988a) to explain a maximum effect that he observed for disjunctions and a minimum effect that he observed for conjunctions (Hampton, 1988b). In those studies, Hampton used either between-subject designs, in which simple sentences, conjunctions, and disjunctions were rated by different groups of participants, or blocked designs, in which each participant rated all types of sentences but in different blocks of trials or experimental sessions. According to his anchoring hypothesis, in order to use the entire range of the response scale, participants use a stricter criterion when judging whether an exemplar is a member of a disjunctive category (contraction) and a looser criterion when judging whether an exemplar is a member of a conjunctive category (expansion). Use of a stricter criterion is akin to contracting definitions; use of a looser criterion is akin to expanding them.

Although it is perhaps intuitively clear that contraction would decrease the truth of disjunctions and expansion would increase the truth of conjunctions, it is not immediately obvious whether reasonable amounts of contraction and expansion are sufficient to produce the maximum and minimum effects. Accordingly, a version of the multiple definitions model that included an expansion and contraction component was simulated, and the behavior of this simulated model observed.

The simulation determined the truths of simple statements as well as of conjunctions and disjunctions by sampling multiple definitions (for the results presented here, the number was fixed at 100) of middle-aged. The central age (e.g., in the definition 40–50 years old, the central age would be 45 years old) of each definition and the width of the definition were determined by sampling from normal distributions. For the results presented here, the mean of the distribution of central ages was set to 45 and its standard deviation varied from 2 to 5 to 8 across different runs of the simulation. The mean of the distribution of the width of definitions varied across different runs of the simulation from 10, 12, 15, 18, to 20 years; its standard deviation was defined as a proportion of the mean. Across different runs, that proportion varied from 0.2 to 0.5 to 1.0. The amount of expansion (i.e., the expansion factor) of each definition for conjunctions and of contraction (i.e., the contraction factor) for disjunctions was fixed for a given run of the simulation and varied across 0, 1, 2, and 3 years across runs. An expansion factor of *n* means that a sampled definition was extended by *n* years on each end (e.g., if the sampled definition were 39–51 and the expansion factor 1, the expanded definition used in computing the truth of a conjunction would be 38–52). Similarly, if the contraction factor for disjunctions was 1, and the sampled definition was 39–51, then the contracted definition used to evaluate the truth of a disjunction would be 40–50. All of the above described values of the different parameters were combined factorally across different runs of the simulation. Each run produced results for 100 participants.

The simulation determined the truth of a statement (e.g., “A person is middle-aged at both 35 and 53”) by sampling 100 definitions and determining the proportion that met the criterion specified by the statement (in the example, the proportion that included both 35 and 53), after expanding (for conjunctions) or contracting (for disjunctions) the definitions by the appropriate amount. Hence, truth values computed by the simulation were in the interval [0,1].

**FIGURE 3:**
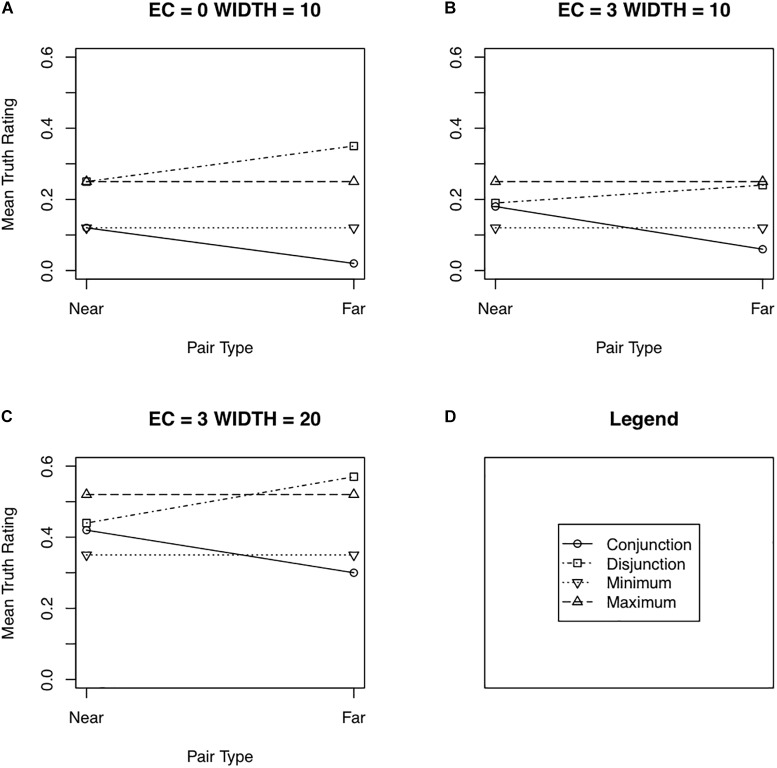
Selected results from the simulations of the contraction–expansion hypothesis showing mean truths as a function of pair type (near or far). EC is the expansion–contraction factor in years. WIDTH is the mean width in years of the sampled definitions. **(A–C)** In all three shown cases, the mean central age of the sampled definitions was 45 and its standard deviation 5 years. The standard deviation of the width of the sampled ages was ½ of WIDTH. See the text for additional details. Panel **(D)** shows the legend used in Panels **(A)** through **(C)**.

Figure 3 shows the results of the simulation for some selected parameter values. The entire set of results of the simulations can be summarized as follows:

(1) Distance effects for conjunctions: With a small standard deviation of the distribution of the central age of the definitions, distance effects for conjunctions were weak (on the order of 2–4%) across all the simulated distributions varying the width of the definitions, partly because the truth of conjunctions tended to be near 0. At intermediate values of the standard deviation of the distribution of the central age, robust distance effects emerged, from 10 to over 15%; at large standard deviations, distance effects were even larger, often reaching nearly 25%. These distance effects tended to increase with the mean width of the definitions and to decrease with the standard deviation of that width. However, over the values tested here, the effects remained robust. The expansion factor had little influence on the distance effects for conjunctions.
(2) Distance effects for disjunctions paralleled those for conjunctions. They were weak at a small standard deviation of the central age (2–4%), stronger for an intermediate standard deviation (9 to over 15%) and stronger still for the large standard deviation (from just under 15% to nearly 25%). As was the case for conjunctions, distance effects for disjunctions increased with increasing mean width of definitions and decreased somewhat with increasing standard deviations of the mean width. They were little affected by the contraction factor.
(3) Minimum effects for near conjunctions did not of course occur with an expansion factor of 0. They did emerge with an expansion factor as small as 1, but did not exceed 7% until an expansion factor of 3 was used. They decreased with increasing standard deviation of the central age distribution (the opposite direction observed for distance effects). They tended to increase with increasing mean width of definitions when the standard deviation was low or intermediate, but to decrease when the standard deviation was high.
(4) Maximum effects for near disjunctions also began to emerge with a contraction factor of 1, but remained weak until a contraction factor of 2 or 3 was used. Maximum effects tended to be slightly larger than minimum effects, but were affected in similar ways by the standard deviation of the central age, the mean width of definitions, and the standard deviation of the width of the definitions.

Overall, the results of the simulations indicate that distance effects, perhaps not surprisingly, occur over a wide range of the parameter values tested here. In addition, robust minimax effects occur with a sufficiently large amount of expansion/contraction, on the order of 2–3 years on both ends of the definition. That overall pattern of results suggests that the expansion/contraction hypothesis is a viable contender to explain the minimax effect.

Another result of Experiments 1 and 2, however, was difficult to reproduce in the simulations. In the experiments, a maximum effect occurred not only for near disjunctions but also for far disjunctions (although the effect was not statistically significant in Experiment 1). The simulations also produced a maximum effect for far disjunctions, but only at the lowest value tested for the variance of the central age of sampled definitions (the same condition that produces only small distance effects). Although it may be premature to reject the expansion–contraction hypothesis on that result alone, the result certainly does suggest that the hypothesis needs to be regarded with a healthy measure of skepticism. Simulations using more extreme expansion–contraction values than used in the results reported above did result in a maximum effect for far disjunctions simultaneously with robust distance effects. However, those parameter values appear to stretch the limits of the psychologically plausible. For instance, with a mean width of definitions of 20 years and a large contraction factor of 10 years, a maximum effect of 18% was produced for far disjunctions. For that same mean width and a contraction factor of 5 years, the maximum effect for far disjunctions was less than 0.5%.

## General Discussion

The primary purpose of this paper was to test a straightforward prediction of the multiple definitions model. That model proposes that certain fuzzy concepts, those that can (at least intuitively) be defined as a range of values over a one-dimensional or perhaps low dimensional space, are represented in the mind as a set of multiple definitions, where each definition is an exact range of values on that dimension. The prediction tested was the distance effect: conjunctions over two exemplars of such a concept should be judged truer when the exemplars are nearer to one another on the dimension over which the concept is defined than when they are farther from one another, controlling for the truths of the individual exemplars. Conversely, disjunctions should be judged as less true when the disjunction involves near pairs as opposed to far pairs. The data from the two experiments reported here offer good support for that prediction, especially for conjunctions, and are hence consistent with the multiple definitions model. To my knowledge, this distance effect has not been previously reported.

The data, however, also showed a minimax effect: near conjunctions were rated as truer than the less true of their two constituents, and disjunctions were rated as less true than the truer of their two constituents. Experiment 2 offered some support for the hypothesis that the minimax effect was the result of some participants using an averaging strategy to determine the truths of conjunctions and disjunctions. However, that support was not as strong as might be desired. Hence, the expansion–contraction hypothesis was offered as another potential (and clearly *post hoc*) explanation of the minimax effect. Although simulations did not conclusively rule out that hypothesis, they did suggest that the amount of contraction for disjunctions needed to predict the maximum effect quantitatively was perhaps larger than what could be considered psychologically plausible. In brief, the multiple definitions model offers a reasonable explanation of the distance effect. A reasonable explanation of the minimax effect, however, is still wanting, as is a model that simultaneously explains both effects.

As mentioned in the “Introduction” section, the multiple definitions model is quite similar to the approach of Kamp and Partee (1995) and its generalization by Douven (2016) and Douven et al. (2017), following Gärdenfors (2000). In terms of the present experiments, these models make the same qualitative predictions as the multiple definitions models, in particular the distance effect. Consequently, the finding of a distance effect is also support for those models. There is, however, at least one difference between those models and the multiple definitions model that is perhaps worthy of mention. The Kamp and Partee (1995), Douven (2016), and Douven et al. (2017) approaches begin with a prototype. With respect to the concept middle-age, for instance, the Kamp and Partee (1995) model would begin with a range of ages in which t(X) = 1 for all ages X in that range, and another set of ages Y for which t(Y) = 0 for all ages in that set Y. In contrast, the multiple definitions model does not necessarily have a prototype. The peak middle age (P) is merely the age contained in the plurality of definitions. There is no requirement that it be contained in all or even nearly all the definitions.^5^ In the present experiment, that distinction is of no consequence. Whether it is indeed important in other contexts (and which approach more accurately describes how humans represent concepts) remains to be determined by future research.

An effect that is at least superficially similar to the minimax effect occurs for conjunctions and disjunctions in the more traditional judgment and reasoning literature. Given a description of Linda as a stereotypical liberal, for example, people are more likely to believe that she is a feminist bank teller than that she is simply a bank teller (Tversky and Kahneman, 1983). This effect has been referred to as the conjunction fallacy. The corresponding disjunction fallacy (Fisk, 2002) is that people are more likely to believe that Linda is active in the feminist movement than to believe that she is either active in the feminist movement or a bank teller.

A similar effect occurs in the conceptual combination literature. Hampton (1988b), for instance, had people rate a single exemplar’s degree of membership in two individual categories, A (e.g., *sports*) and B (e.g., *games*), as well as in a category that was a conjunction of the two, A and B (e.g., *games that are sports*). He found a number of instances where the exemplar was judged to be a member of the conjoined category even though it was not judged to be a member of at least one of the two simple categories. Hampton termed this phenomenon *overextension* (see also Smith and Osherson, 1984). Hampton (1997) and Storms et al. (1999) also found overextension for some exemplars in conjunctions where one of the two constituent categories was negated (e.g., *birds which are not pets*). Aerts et al. (2015) generalized this finding of overextension to all possible pairings of two concepts and their negations: A and B, A and not B, not A and B, and not A and not B (and also discovered other aspects of human judgment data that do not follow the laws of classical probability theory). Hampton (1988a) found an analogous result to overextension for disjunctions. The degree of membership of *tomato*, for example, in the disjunctive category *fruit or vegetable* might be judged to be lower than its maximum degree of membership in the individual constituents. That is, people may be more willing to believe that *tomato* is a *fruit* than to believe that it is either a *fruit or a vegetable*, a phenomenon he termed *underextension*.

The minimum effect, the conjunction fallacy, and overextension are all analogous to one another—the truth of a conjunction is rated to be higher than the minimum truth of its constituents. Likewise, the maximum effect, the disjunction fallacy, and underextension are also analogous. The truth of a disjunction is rated to be lower than the maximum truth of its constituents. There is, however, a subtle difference between the conjunction fallacy and overextension, on the one hand, and the minimum effect on the other hand. The conjunction fallacy and overextension involve the conjunction of a single Subject (e.g., *tomato*) over two Predicates (e.g., *vegetable and fruit*). This emphasis on conjunctions over two predicates makes imminent sense in the conceptual combination literature—after all, the concern is with the combination of two concepts (predicates). The minimum effect, in contrast, involves a conjunction of two Subjects (e.g., *45 and 60*) over a single Predicate (e.g., *middle-age*). (In this respect, the minimax effect, like the distance effect, is a new effect.) Despite this potential difference, it is useful to examine explanations that have been offered for the conjunction fallacy and for overextension in order to determine if they may also apply to the minimum effect. Similar comments apply to the disjunction fallacy and underextension, on the one hand, and the maximum effect, on the other hand.

One approach that has had some success in explaining both overextension and underextension in the conceptual combination literature involves the notion of a composite prototype (e.g., Smith et al., 1988; Huttenlocher and Hedges, 1994; Hampton, 1997, 2007). According to this approach, to determine the degree to which exemplar *x* is a member of category *A*, people determine the similarity of *x* to the prototype of *A*. The degree of membership of *x* in A is then directly proportional to the similarity of *x* to *A*’s prototype. When determining *x*’s degree of membership in the conjoined category *A and B*, however, people do not compare *x* to the prototype of *A*, then to the prototype of *B*, and then determine *x*’s degree of membership in *A and B* by using some function to combine those two similarity measurements, such as the product rule or the minimum rule. Instead, people form a new, composite category from the two constituent categories, and that composite category has its own prototype. That composite prototype may inherit some attributes from category *A*, others from *B*, it may discard other attributes from one or the other of the categories, or it may increase the weight or importance of certain attributes or attribute values in the composite, depending upon the context and precise wording of the conjunction or disjunction. To take an example from Smith et al. (1988) the conjoined concept *red apple*, may inherit attributes from the concept *apple*, but with a particularly large weight attached to the color attribute, and with the values of that attribute limited to *red*, rather than including, say, *red, green*, and *brown*. As mentioned, this approach has been able to successfully explain both overextension in conjunction and underextension in disjunction. Unlike the experiments that gave rise to the hypothesis of a composite prototype, the stimuli in the present experiments involved a single concept (middle-age) rather than two. Consequently, there is no second category with which to form a composite, and the notion of a composite prototype would seem not to be applicable to the present experiments. This comment should in no way be taken to be dismissive of the idea of a composite prototype or to argue that the hypothesis of a composite prototype is somehow wrong. It is simply to point out that the hypothesis does not apply to the present experiment. The notion of a composite prototype may still have an important role in the conceptual combination literature.^6^

Busemeyer et al. (2011) and Busemeyer and Bruza (2012) have recently developed explanations for both the conjunction and disjunction fallacies using principles of quantum probability theory. These explanations would also apply to overextension and underextension. Also in the context of quantum probability theory, but using a somewhat different approach, Aerts (2009); Aerts et al. (2015), and Aerts et al. (2016) have successfully modeled both overextension and underextension, including a number of cases that involve the negation of conjunctions (Sozzo, 2015). As currently formulated, those explanations address the case where the conjunction or disjunction is of a single subject (e.g., Linda) over multiple predicates (e.g., being a feminist/being a bank teller), much like the case for the composite prototype models. Although it is not immediately obvious (at least to the present author) how to extend these models to the case of conjunctions and disjunctions of two subjects over a single predicate, such an extension could well be possible. Indeed, given the success of quantum probability theory to explain a number of anomalous findings in the human judgment literature (Busemeyer and Bruza, 2012), there is reason to be optimistic in this regard. The approach of Aerts et al. (2015, 2016) is particularly intriguing. These authors have argued that human reasoning involves a superposition of two simultaneous process. One concerns what they term “emergent reasoning,” and involves the formation of new, emergent concepts, similar to the notion of composite prototypes discussed above. This emergent reasoning process does not follow the rules of classical logical. The second process, which they term “logical reasoning,” does follow the rules of classical logic. The multiple definitions model does not follow classical logic, in that it violates what Osherson and Smith (1982) termed the SFH—in the multiple definitions model, the truth of a compound sentence is not a function of the truths of its simple constituents. Given its processing model (the sampling of definitions), however, the model does follow the rules of classical probability theory. It is the sampling process that allows the model to successfully predict the distance effect. However, the model fails to predict the minimax effect, and that effect is also at odds with classical probability theory. Conceivably, within the context of the approach of Aerts et al. (2015, 2016), the minimax effect reflects the emergent reasoning process and the distance effect reflects the logical reasoning process. In any event, a model that was able to simultaneously predict both effects would be a striking success.

### Relation to Other Models of Categorization

The multiple definitions model is intended at least as much as a model of concept representation as it is of human reasoning. As such, a few comments on how it relates to other models of categorization are in order. The relation of the multiple definitions models to the work of Kamp and Partee (1995), Gärdenfors (2000), Decock and Douven (2014), Douven (2016), and Douven et al. (2017) has already been mentioned. More generally, perhaps the two most dominant approaches to categorization in the psychological literature are prototype models and exemplar models. In exemplar models, the category is represented as the set of its exemplars or members (Medin and Schaffer, 1978; Nosofsky, 1986, 2011). A new exemplar is assigned to a category to the extent that it is more similar to the exemplars of that category than to exemplars of other possible categories. Exemplars, in turn, are represented as points in space. Typically, in experiments testing these models, the stimuli are varied along a set of pre-defined dimensions, such as size, hue, and shape. Those dimensions define the dimensions of the space, and the particular values a given stimulus has on those dimensions then defines a point in that space. Spatial models of semantic memory (Landauer and Dumais, 1997; Jones and Mewhort, 2007) represent word meanings in a similar fashion—a word’s meaning is defined as a point in a space with typically several hundred dimensions.

From the perspective of assumptions concerning the representation of concepts, the multiple definitions model is certainly within the spirit of exemplar models, where the definitions serve as exemplars. In fact, the model can be viewed as a natural extension of models such as Nosofsky’s (1986) Generalized Context Model (GCM), which is most frequently used to model how single exemplars are mapped into different categories, to situations where conjunctions, disjunctions, and perhaps other groupings of multiple exemplars are mapped to a single category. One difference is that the definitions are not a single point, as they most commonly are in the GCM, but a range over a space. Intuitively, for the types of concepts that inspired the multiple definitions model, ranges seem to make more sense than do points. Whether traditional exemplar models of categorization, such as the GCM, or spatial models of semantic memory, could benefit by conceiving of exemplars as regions of space rather than a point in space is a question for further work. In the context of the present study, a fair question to ask is whether the definitions must consist of a range of ages, as opposed to a single age (i.e., a single point in space), in order to produce the distance effect. I examined a “point-definition” model in which sampled definitions consisted of a single age rather than a range of ages. In the model, t(X) is inversely proportional to the mean distance of X to each sampled age. t(X ^∧^ Y) is inversely proportional to the mean of the mean distance of X and Y to each sampled age. t(X ∨ Y) is inversely proportional to the mean of the minimum distance of X and Y to each sampled age. Those definitions are reasonable definitions of conjunction and disjunction, respectively, in such a model. The model does produce a distance effect for disjunctions (but not a maximum effect). It does not, however, produce a distance effect for conjunctions (though it does produce a minimum effect).

From the perspective of processing assumptions, there may be a more substantial difference between the multiple definitions model and traditional exemplar models. Exemplar models rely heavily on similarity—an exemplar’s similarity to other category members determines the likelihood that that exemplar is included in the category. Similarity has no such overt role in the multiple definitions model. While it is true that a given definition may likely contain exemplars that are similar to one another, determination of category membership does not require any computations of similarity.

In prototype models, a category is represented by a single prototype, which reflects the central tendency across all category exemplars along all dimensions on which the exemplars vary (Reed, 1972; Minda and Smith, 2011). Like exemplars in exemplar models, the prototype in most models is a point in space. More recent models have allowed for a category to be represented by multiple prototypes (or clusters), rather than a just a single prototype (e.g., Rosseel, 2002; Love et al., 2004; Vanpaemel and Storms, 2008; McDonnell and Gureckis, 2011). Each such prototype is again typically a point in space. If each definition in the multiple definitions model is considered as a prototype, then the multiple definitions model could perhaps be considered as such a multiple-prototype model, where each prototype is a range rather than a point. The multiple definitions model, though, does seem to violate the spirit of multiple-prototype models. Such models were motivated to a large extent to accommodate within prototype theory non-linearly separable categories, or cases where different subsets of a category’s exemplars cluster in very different areas of the category space and hence, across subsets, can be dissimilar to one another. These criteria do not apply to concepts such as “middle-age” or “Midwestern city” that inspired the multiple definitions model.

Two concluding comments are in order. First, it is perhaps obvious from the above discussion that although the multiple definitions model was framed in the introduction as applying to one-dimensional (middle-age) or low dimensional (Midwestern city) concepts, there is nothing inherent in the model that would prevent it from being applied to concepts defined in higher dimensional spaces. If a concept were defined in an *n*-dimensional space, a definition would simply be an *n*-dimensional volume from that space instead of a range over the one-dimensional line of ages.

Finally, it should be noted that the multiple definitions model is described here as if the definitions exist in the mind. They are the category representation. For purposes of the present experiments, an equivalent approach is that the category representation is not a set of definitions, but a set of probability distributions for generating the definitions.^7^ The definitions are then generated on an as-needed basis and discarded when no longer needed. Discriminating between these two alternatives is again a question for future work.

## Ethics Statement

This study was carried out in accordance with the recommendations of and approval of the Indiana University Institutional Review Board with written informed consent from all subjects. All subjects gave written informed consent in accordance with the Declaration of Helsinki. The protocol was approved by the Indiana University Institutional Review Board.

## Author Contributions

TG has made a substantial contribution to the conception and design of the work and also contributed to the data acquisition, analysis and interpretation of the data, drafting the manuscript and revising it critically for important intellectual content, and approved the final version to be published.

## Conflict of Interest Statement

The author declares that the research was conducted in the absence of any commercial or financial relationships that could be construed as a potential conflict of interest.

## Footnotes

1 In other words, the concept of middle-age forms a convex region, as defined by Gärdenfors (2000). Here, for clarity, the assumptions were phrased as applied to the one-dimensional concept of age. Gärdenfors generalizes this approach to spaces of higher dimensionality. Gärdenfors argues that most concepts are represented by humans in the mind as such convex regions. The present paper is neutral with respect to that claim. What is necessary for the present paper is to work with a concept(s) for which commonsense dictates that it is extremely likely that the representation in the mind is a convex region.

2 The unit information Bayes factor is one method of calculating the likelihood ratio of the alternative to the null hypothesis (or, reciprocally, the null to the alternative) (Rouder et al., 2009). Hereafter, this measure is referred to simply as the Bayes factor. In calculating the Bayes factor, the default value of the parameter *r* = 0.707 was used as suggested by Rouder et al. (2009), as I had no *a priori* reason to suspect a particularly large or particularly small effect size.

3 The minimum effect reported here for conjunctions should not be confused with the well-known conjunction fallacy (Tversky and Kahneman, 1983) in the judgment literature or the minimum effect described in the conceptual combination literature (Osherson and Smith, 1982). In the present case, the conjunction is across two subject terms and involves a single predicate: S1 and S2 are both P. In the conjunction fallacy and in the conceptual combination literature the conjunction is across two predicates and involves a single subject term: S is both P1 and P2. Whether the two effects are due to the same psychological mechanisms is an empirical question. This point is discussed further in the “General Discussion” section.

4 The original plan was to recruit 80 participants. Based on the results of Experiment 1, the hope was that 60 of those participants would have shown equal pairs as defined in connection with Experiment 1. In an analysis that then included those 60 participants (i.e., before dividing them into Logical and Averaging participants), the power to detect a distance effect for disjunctions (That effect in Experiment 1 was smaller than the distance effect for conjunctions), that was the same size as that found in Experiment 1, was 0.80. Unfortunately, a much smaller proportion of participants showed equal pairs in Experiment 2 than in Experiment 1 and the goal of 60 was not reached. Consequently, additional participants were recruited until the participant pool became unavailable due to the end of the academic year, but the goal of 60 was still not reached.

5 In terms of the multiple definitions model, a prototype can be considered an emergent property—those concepts which do have prototypes are concepts in which it turns out that all or nearly all definitions include one (perhaps several) particular potential exemplar. That exemplar is then the prototype.

6 It may be worth noting here that, although extending the multiple definitions approach to conceptual combinations is well beyond the scope of the present paper, there are ways to make those extensions. In order to work empirically, in particular, in order to predict overextension and underextension, the most obvious such extensions would likely require some sort of idea akin to composite prototypes—composite definitions in the case of the multiple definitions model.

7 In fact, exactly this approach was taken in the simulations of the contraction–expansion hypothesis.
